# Re-Irradiation with Intensity-Modulated Radiation Therapy for the Treatment of Recurrent Cervical Cancer in the Pelvis: An Analysis of Outcomes and Toxicity

**DOI:** 10.3390/medicina59061164

**Published:** 2023-06-17

**Authors:** Hye Jin Kang, Yoo-Kang Kwak, So Jung Lee, Myungsoo Kim

**Affiliations:** Department of Radiation Oncology, Incheon St. Mary’s Hospital, College of Medicine, The Catholic University of Korea, Seoul 06591, Republic of Korea; jade2959@gmail.com (H.J.K.); behappy1219@catholic.ac.kr (Y.-K.K.); sunport@naver.com (S.J.L.)

**Keywords:** cervical cancer, recurrence, salvage therapy, intensity-modulated radiation therapy, re-irradiation

## Abstract

*Background and Objectives*: Treatment options for most patients with recurrent cervical cancer within the previously irradiated field are limited. This study aimed to investigate the feasibility and safety of re-irradiation using intensity-modulated radiation therapy (IMRT) for patients with cervical cancer who experienced intrapelvic recurrence. *Materials and Methods*: We retrospectively analyzed 22 patients with recurrent cervical cancer who were treated with re-irradiation for intrapelvic recurrence using IMRT between July 2006 and July 2020. The irradiation dose and volume were determined based on the range considered safe for the tumor size, location, and previous irradiation dose. *Results*: The median follow-up period was 15 months (range: 3–120) and the overall response rate was 63.6%. Of the symptomatic patients, 90% experienced symptom relief after treatment. The 1- and 2-year local progression-free survival (LPFS) rates were 36.8% and 30.7%, respectively, whereas the 1- and 2-year overall survival (OS) rates were 68.2% and 25.0%, respectively. Multivariate analysis revealed that the interval between irradiations and gross tumor volume (GTV) were significant prognostic factors for LPFS. The response to re-irradiation showed borderline statistical significance for LPFS. The GTV and response to re-irradiation were also independent prognostic factors for OS. Grade 3 late toxicities were observed in 4 (18.2%) of the 22 patients. Recto- or vesico-vaginal fistula occurred in four patients. The irradiation dose was associated with fistula formation with borderline significance. *Conclusions*: Re-irradiation using IMRT is a safe and effective treatment strategy for patients with recurrent cervical cancer who previously received RT. Interval between irradiations, tumor size, response to re-irradiation, and radiation dose were the main factors affecting efficacy and safety.

## 1. Introduction

Cervical cancer is the fourth most common malignancy in women based on both incidence and mortality, although its incidence is declining due to advances in prevention and screening. Approximately 570,000 new cases and 310,000 deaths occur worldwide annually [1]. Radiation therapy (RT) is generally used as a primary treatment for cervical cancer because it responds well to radiation [2]. Overall, approximately 80% of patients with cervical cancer receive RT [3]. Patients receiving definitive RT have a favorable prognosis, with a 5-year overall survival (OS) of 80–90% for early-stage disease and 60–70% for advanced-stage disease [4,5]. After initial treatment, about 30–40% of patients experience disease recurrences and 50% of these are loco-regional recurrences within the conventional pelvic irradiation field [6,7]. Sommers et al. reported that the 5-year OS rate for patients with recurrent cervical cancer within the previously irradiated field was only 1% if they did not receive any treatment [8].

The National Comprehensive Cancer Network guidelines recommend pelvic exenteration or radical hysterectomy in cases of central disease and individualized re-irradiation or resection in cases of noncentral disease [9]. Although pelvic exenteration is effective in patients with no pelvic wall involvement and distant metastasis, pelvic removal is generally avoided because of the difficulties associated with surgical interventions on previously irradiated tissue [10]. Re-irradiation treatment is not a generally accepted treatment for patients with previous pelvic irradiation because it is believed to be associated with severe late toxicity [11]. However, due to technological advancement, re-irradiation has garnered increased attention from clinicians in recent years. In particular, intensity-modulated RT (IMRT) can deliver various intensified radiation doses within the treatment field using multi-leaf collimators and an inverse planning system. Consequently, this technology has enabled the delivery of conformal radiation doses at the target volumes while reducing the dose to adjacent critical structures [12]. Increasing evidence for pelvic re-irradiation with IMRT supports its favorable efficacy and safety in various recurrent cancers, including rectal and prostate cancers [13,14]. Nevertheless, re-irradiation of recurrent cervical cancer using IMRT remains challenging.

We aimed to investigate the feasibility and safety of re-irradiation using IMRT for patients with cervical cancer who experienced intrapelvic recurrence after previous pelvic irradiation at our hospital. Moreover, we identified patients who would receive the most clinical benefit from re-irradiation by determining the prognostic factors.

## 2. Materials and Methods

### 2.1. Patients

Between July 2006 and July 2020, a total of 507 patients with cervical cancer who received RT at our institution were evaluated. The inclusion criteria were as follows: (1) histologically proven primary cervical cancer; (2) previous treatment with whole pelvic RT with definitive or adjuvant intent; and (3) treatment of intrapelvic recurrence with re-irradiation using IMRT. The exclusion criteria were as follows: (1) treatment with stereotactic body RT (SBRT); (2) previous history of other malignancies; and (3) no follow-up after re-irradiation treatment. Of the 36 patients who received re-irradiation, 14 were excluded and 22 were included in the study. This study was approved by the Institutional Review Board of our institution (Incheon St. Mary’s Hospital, The Catholic University of Korea; reference number: OC22RASI0113). All data were retrieved from medical reports and our institutional medical records.

### 2.2. Treatment

Contrast computed tomography (CT) was performed for target delineation. Gross tumor volume (GTV) was defined as a recurrent tumor on CT imaging. Clinical target volume (CTV) was defined as GTV plus 5–10 mm margins for microscopic tumor spread, and the CTV was modified to reduce the re-irradiation dose to nearby organs at risk (OARs). A 3.0 mm expansion from the CTV was applied to the planning target volume (PTV), considering patient motion and daily setup errors.

The administered dose for re-irradiation was determined based on the range considered safe, taking into account both toxicity and tumor control. All patients were re-irradiated with helical fan-beam IMRT using the Hi-Art Tomotherapy system (TomoTherapy Inc., Madison, WI, USA). The goal was to ensure that >95% of the PTV received 95% of the prescribed dose. Treatment planning was designed to minimize the dose to the OARs; however, no specific dose constraints were used for treatment planning.

The cumulative dose of radiation was calculated as the sum of the total dose of the previous RT and the re-irradiation dose. To account for differences in dose and fractionation, the doses were converted to equivalent doses in 2 Gy/fraction (EQD2) using the linear quadratic model, with an α/β ratio of 10.

### 2.3. Follow-Up and Response Evaluation

For most cases, the first follow-up was conducted 1 month after radiotherapy. Subsequently, patients were followed up every three months for the first two years, every six months until five years, and annually thereafter. The standard follow-up surveillance program consisted of taking a clinical history, physical examination, a Papanicolaou test, and laboratory work-ups, including squamous cell carcinoma (SCC) antigen assay, and radiographic imaging with CT and/or pelvic magnetic resonance imaging.

Treatment response to re-irradiation was evaluated using the Response Evaluation Criteria in Solid Tumors version 1.1. A complete response (CR) was defined as the disappearance of the entire target lesion and a partial response (PR) was defined as at least a 30% reduction in the sum of the longest diameter of the target lesion. Progressive disease (PD) was defined as at least a 20% increase in the sum of the longest diameter of the target lesion. Stable disease (SD) was defined as when none of the above applied. The response rate represented the proportion of patients who achieved CR or PR. Treatment-related toxicity was investigated based on the Common Terminology Criteria for Adverse Events, version 5.0. Toxicities occurring within/after 90 days of RT completion were considered acute/late toxicities, respectively.

### 2.4. Statistical Analyses

The Kaplan–Meier method was used to estimate local progression-free survival (LPFS) and OS. LPFS was defined as the duration from the last day of re-irradiation treatment to the date of in-field progression, death from any cause, or last follow-up visit for patients free of disease. OS was defined as the time from the last day of re-irradiation treatment to death from any cause or last follow-up visit. Univariate analyses were performed using the Cox regression model to assess the prognostic factors associated with local progression or survival. Potential prognostic factors with *p* < 0.1 in the univariate analyses were included in multivariate analyses. Multivariate analyses were conducted using the Cox proportional hazards model. All test results were two-sided. Statistical significance was set at *p* < 0.05. All statistical analyses were performed using the R software, version 4.2.1.

## 3. Results

### 3.1. Patient and Tumor Characteristics

Baseline patient and disease characteristics are summarized in Table 1. The median age at the time of recurrence was 52 years (range: 29–75). At initial diagnosis, all patients had Eastern Cooperative Oncology Group (ECOG) performance status of grades 0–1. However, four patients were identified with ECOG performance status of grade 2 at the time of recurrence. The main histological type was SCC (*n* = 20, 90.9%), and the remaining patients had adenocarcinoma (*n* = 2, 9.1%). Half of the patients had International Federation of Gynecology and Obstetrics (FIGO) stage IIIC disease with pelvic and/or para-aortic lymph node involvement at the time of initial diagnosis.

A total of 12 (54.5%) patients received definitive pelvic RT, and 10 (45.5%) patients underwent pelvic RT as adjuvant therapy after surgery. A total of 12 (54.5%) patients received whole pelvic RT alone, and 10 (45.5%) patients received whole pelvic RT with hypofractionated boost RT using an external beam RT or brachytherapy.

### 3.2. Treatment after Recurrence

Disease and treatment characteristics after recurrence are shown in Table 2. The median interval from the end of primary RT to the diagnosis of recurrence was 10.5 months (range: 2–1354). Recurrences occurred at the primary sites only, such as the cervix or vaginal stump in 9 (40.9%) patients, at the pelvic lymph node in 3 (13.7%) patients, at the primary site and pelvic lymph node in 5 (22.7%) patients, and at the primary site or pelvic lymph node with distant metastasis in 5 (22.7%) patients.

**Figure 1:**
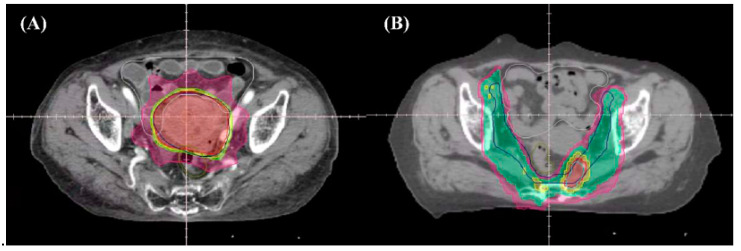
Example of re-irradiation plans: (**A**) A 74-year-old woman underwent re-irradiation therapy with palliative intent for recurrent cervical mass. The re-irradiation dose was 30 Gy in 10 fractions (navy line = PTV; red line = 30 Gy; yellow line = 27 Gy; green line = 24 Gy; purple line = 18 Gy). (**B**) A 65-year-old woman underwent re-irradiation therapy with salvage intent for pelvic lymphatics including recurrent lymph node. The re-irradiation dose was 55 Gy for recurrent mass (PTV1) and 45 Gy for lymphatics (PTV2) in 25 fractions (aqua line = PTV1; navy line = PTV2; red line = 55 Gy; yellow line = 50 Gy; green line = 45 Gy; purple line = 40 Gy).

Only one patient underwent salvage surgery for regional lymph node recurrence before undergoing re-irradiation. A total of 13 (59.1%) patients received salvage chemotherapy (before re-irradiation: 10, concurrent with re-irradiation: 3). The median interval between primary treatment and re-irradiation was 23 months (range: 3–1360). Re-irradiation was performed with salvage intent in 15 (68.2%) patients and palliative intent in the remaining 7 (31.8%) patients. The re-irradiation fields were typically limited to the recurrent mass in 17 patients. The fields in the remaining five patients were expanded to include the recurrent mass with the involved lymphatic chain. An example of re-irradiation treatment field is shown in Figure 1. The median GTV was 99.8 cm^3^ (range: 7–226.5). The median re-irradiation dose to the recurrent site was 50 Gy (range: 26–56). The median cumulative dose from the previous RT and re-irradiation course was 110 Gy (range: 83–148).

### 3.3. Treatment Outcomes

Planned re-irradiation treatment was completed in all the patients and the median follow-up period was 15 months (range: 3–120). A total of 7 (31.8%) patients achieved CR, 7 (31.8%) patients achieved PR, and 5 (22.7%) patients had SD. Accordingly, the overall response rate was 63.6%. Of 22 patients, half had identifiable symptoms after recurrence: 7 patients experienced pain and 4 experienced vaginal bleeding. Approximately 90% (10/11) experienced symptom relief after treatment. The median duration of symptom relief was 6 months (range: 3–49) and five patients experienced permanent symptom relief.

A total of 14 (63.6%) patients exhibited local progression at a median of 12 months. The 1- and 2-year LPFS rates were 36.8% and 30.7%, respectively. At the time of analysis, a total of 19 patients had died: 10 (52.6%) had died of local progression, 6 (31.6%) had died of metastases, and 3 (15.8%) had died of non-tumor causes. The median OS was 16 months, and the 1- and 2-year OS rates were 68.2% and 25.0%, respectively.

### 3.4. Prognostic Factors Associated with LPFS and OS

The factors that may be associated with survival outcomes after re-irradiation were analyzed. Age (<50 years vs. ≥50 years), ECOG performance status at recurrence (0–1 vs. 2), histologic type, initial FIGO stage (I/II vs. III/IV), progression-free interval (<10 months vs. ≥10 months), SCC antigen at recurrence (1.55 ng/mL vs. ≥1.55 ng/mL), chemotherapy at recurrence, interval between the primary treatment and re-irradiation (<6 months vs. ≥6 months), purpose of re-irradiation, presence of disease related symptom, GTV (<50 cm^3^ vs. ≥50 cm^3^), involved lymphatic irradiation, dose of re-irradiation (<50 Gy vs. ≥50 Gy), cumulative RT dose (<110 Gy vs. ≥110 Gy), and response to re-irradiation (CR/PR vs. SD/PD) were included in the analysis. The detailed analysis results of the prognostic factors for LPFS and OS are shown in Table 3.

**Figure 2:**
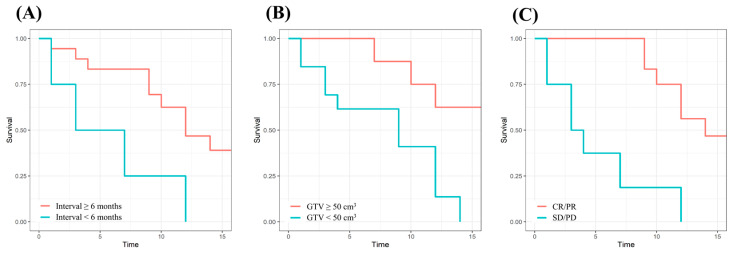
Kaplan–Meier curves showing local progression-free survival based on (**A**) interval between irradiations, (**B**) gross tumor volume size, and (**C**) response to re-irradiation.

Re-irradiation interval (*p =* 0.026), GTV (*p =* 0.015), and re-irradiation response (*p =* 0.001) were significantly associated with LPFS in the univariate analysis. The 1-year LPFS rate for patients with re-irradiation interval ≥6 months and response to re-irradiation were 46.9% and 56.2%, respectively. However, all patients with re-irradiation interval <6 months or no response to re-irradiation exhibited progression within 1 year. Large target volumes were associated with poor local control. The 1-year LPFS rates of patients with GTV < 50 cm^3^ and GTV ≥ 50 cm^3^ were 62.5% and 13.7%, respectively (Figure 2). GTV (hazard ratio (HR): 7.31, 95% confidence interval (CI): 1.4–38.22; *p =* 0.018) and re-irradiation interval (HR: 0.11, 95% CI: 0.02–0.60; *p =* 0.010) remained strong predictors of LPFS after multivariate analysis. Response to re-irradiation also exhibited borderline statistical significance (HR: 0.28; 95% CI: 0.07–1.08; *p =* 0.065).

**Figure 3:**
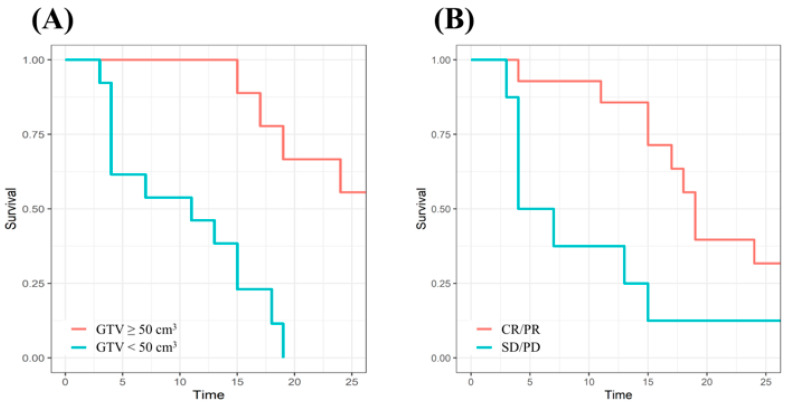
Kaplan–Meier curves showing overall survival based on (**A**) gross tumor volume size and (**B**) response to re-irradiation.

Regarding the prognostic factors for OS, ECOG performance status (*p =* 0.019), GTV (*p =* 0.002), and response to re-irradiation (*p =* 0.014) were significantly associated with OS in the univariate analysis. The GTV (HR: 8.71, 95% CI: 2.11–35.88; *p =* 0.003) and response to re-irradiation (HR: 0.35; 95% CI: 0.12–0.96; *p =* 0.042) were the independent prognostic factors influencing OS in the multivariate analysis. In terms of GTV, the median OS for patients who had small-sized GTVs was 31 months compared to 11 months for those with large-sized GTVs. The 1- and 2-year OS rates were 88.9% and 55.6%, respectively, for patients with GTV < 50 cm^3^ compared to 46.2% and 0% for patients with GTV ≥ 50 cm^3^. Patients who responded to re-irradiation exhibited a higher median OS (19 months) than patients who were resistant to re-irradiation (5.5 months). The 1- and 2-year OS rates for patients who responded to re-irradiation were 85.7% and 31.7%, respectively. In contrast, the 1- and 2-year OS rates for patients resistant to re-irradiation were 37.5% and 12.5%, respectively (Figure 3).

### 3.5. Toxicity

Acute toxicity of re-irradiation was within acceptable levels. A total of 9 (40.9%) patients experienced grade 2 acute toxicity (gastrointestinal: 3, genitourinary: 3, perineal edema: 2, and hematologic: 1). No acute toxicities of grade ≥3 were observed.

A total of 11 patients (50.0%) experienced grade ≥2 late toxicity (grade 2: 7 (31.8%), grade 3: 4 (18.2%)). Some patients had multiple late toxicities. Genitourinary toxicity was observed in five patients, including grade 2 cystitis in three patients and grade 3 ureteral stricture in two patients. Grade 2 perineal edema was also identified in three patients. Grade 2 abdominal pain and grade 2 vaginal bleeding were observed in one patient each. Finally, recto- or vesico-vaginal fistula occurred in four patients, including grades 2 and 3 in two patients each. No grade 4 or treatment-related deaths were observed.

We analyzed the relationship between fistula formation and treatment-related factors. The irradiation dose was associated with fistula formation with borderline significance. The mean cumulative EQD2 of the previous RT was significantly higher in patients with fistula formation (82.2 ± 22.0 Gy vs. 62.7 ± 16.0 Gy; *p =* 0.051). The EQD2 of re-irradiation was also associated with fistula formation (68.2 ± 18.6 Gy vs. 52.2 ± 13.3 Gy; *p =* 0.054).

## 4. Discussion

The RT modalities for re-irradiation in intrapelvic recurrence of cervical cancer are mainly limited to brachytherapy and SBRT. Previous studies reported a response rate of 50–70% for patients with recurrent cervical cancer, who were salvaged with either SBRT or brachytherapy. Additionally, the 1-year LPFS and OS rates were 50–65% and 50–70%, respectively [3,15,16,17,18]. Brachytherapy is currently the most widely used RT modality for recurrent cervical cancer, but it is mainly used for central pelvic recurrence and requires an invasive procedure [3]. High-dose SBRT results in an ablative effect similar to surgery [19]. However, SBRT may also only be considered in patients with limited conditions, such as those with small-sized tumors on the pelvic side wall [20]. In contrast, IMRT could be used more widely, regardless of the location or extent of the tumor.

Few clinical studies have reported the role of IMRT in the intrapelvic recurrence of cervical cancer after previous pelvic irradiation. In our study, re-irradiation with IMRT achieved an overall response rate of 63.6%. The 1-year LPFS rate was 36.8%, and median LPFS time was 12 months. The 1-year OS rate was 68.2%, and the median OS time was 17 months. It is difficult to directly compare these findings to those of previous studies because the study cohorts were heterogeneous in terms of disease extent, tumor size, and RT techniques. In addition, patients with poor performance status, patients who received re-irradiation for palliative intent, and patients with bulky masses were included in our study. Nevertheless, the treatment outcomes are comparable to those reported in previous studies.

Furthermore, we identified patients who would receive the greatest clinical benefit from re-irradiation. Multivariate analysis revealed that the interval between irradiations and GTV were significant prognostic factors for LPFS. The response to re-irradiation showed borderline statistical significance for LPFS. This finding might reflect insufficient statistical power owing to the small number of cases and the heterogeneous nature of the study group. GTV and response to re-irradiation were also independent prognostic factors for OS. These findings were consistent with those of previous studies on prognostic factors for recurrent cervical cancer. In particular, tumor size was confirmed as a strong predictor of local control and survival in numerous studies [16,19,21,22]. Zolciak-Siwinska et al. also demonstrated that a short interval between the initial treatment and re-irradiation and large tumor size were significant prognostic factors adversely affecting LPFS and OS [23]. Response to re-irradiation treatment was also identified as a prognostic factor that strongly influenced the LPFS and OS in recurrent cancer [24,25]. Therefore, re-irradiation treatment could be effective in patients with a prolonged time interval between irradiations, low tumor burden, and favorable response to re-irradiation.

The late grade ≥3 toxicity rate described in previous studies of re-irradiation with brachytherapy was approximately 30% [26,27]. However, Ling et al. found that grade ≥3 late toxicities occurred in 15% of patients who received SBRT for loco-regional recurrences [28]. In the current study of re-irradiation using IMRT, grade 3 late toxicities were observed in 4 (18.2%) of the 22 patients. These results suggest that precisely planned RT using advanced technology improves the safety of re-irradiation. In particular, as few studies have investigated re-irradiation using IMRT, our findings provide useful information, highlighting that re-irradiation with IMRT is a relatively safe treatment option for patients with intrapelvic recurrence of cervical cancer after prior pelvic irradiation. Additionally, four patients in the current study had recto- or vesico-vaginal fistulas of grades 2–3. There was a tendency to increase the radiation dose in patients with fistula formation. Although the upper limit dose that can prevent fistula formation cannot be confirmed in this study, the re-irradiation dose must be determined in consideration of this. This finding may form the basis of future studies to assess the range of re-irradiation doses that are considered safe.

This study had some limitations in addition to its retrospective nature. Selection bias might have been introduced by the physicians when choosing the salvage treatment modality. Furthermore, the data included heterogeneous patients. The patients received a variety of treatments at the time of initial diagnosis and after recurrence, and this could have affected the treatment outcomes in this study. In addition, patient symptoms and treatment-related toxicities might not have been fully documented in medical records and might, therefore, have been underestimated. Lastly, this study included a relatively small number of patients, and this could have impacted the generalizability of the results. Despite the limitations, this study suggests the feasibility of re-irradiation using IMRT for intrapelvic recurrent cervical cancer.

## 5. Conclusions

In summary, re-irradiation using IMRT is a safe and effective treatment strategy for patients with recurrent cervical cancer. It can improve the quality of life of patients by relieving tumor-related symptoms. In addition, our findings suggest that interval between radiations, tumor size, response to re-irradiation, and RT dose are practical considerations that could aid clinical decision-making as factors indicative of treatment efficacy and safety. With further follow-up and more experience, we anticipate reporting more favorable results in the future.

## Figures and Tables

**Table 1:** Patient, disease, and previous treatment characteristics.

Characteristics	N (Total = 22)	%
Age at time of re-irradiation, years		
Median (range)	52 (29–75)	-
ECOG at time of re-irradiation		
0–1	18	81.8
2	4	18.2
Histology		
SCC	20	90.9
Adenocarcinoma	2	9.1
2018 FIGO stage at diagnosis		
IB1	3	13.6
IB2	2	9.0
IIA1	1	4.6
IIB	1	4.6
IIIB	1	4.6
IIIC1	5	22.7
IIIC2	6	27.3
IVA	2	9.0
Unknown	1	4.6
Prior surgery		
Yes	10	45.5
No	12	54.5
Prior chemotherapy		
Sequential	6	27.3
Concurrent	11	50.0
None	5	22.7
Prior pelvic radiotherapy type		
Whole pelvis only	12	54.5
Boost with EBRT	4	18.2
Boost with brachytherapy	6	27.3
Radiation dose of whole pelvis (Gy)		
Median (range)	50.4 (43.2–59.4)	-
Cumulative dose of prior radiotherapy (EQD2, Gy)		
Median (range)	61.1 (42.5–99.2)	-

ECOG, Eastern Cooperative Oncology Group; SCC, squamous cell carcinoma; FIGO, International Federation of Gynecology and Obstetrics; EBRT, external beam radiation therapy; EQD2, equivalent doses in 2 Gy/fraction.

**Table 2:** Disease and treatment characteristics after recurrence.

Characteristics	N (Total = 22)	%
Progression-free interval (months)		
Median (range)	10.5 (2–1354)	-
<10 months	10	45.5
≥10 months	12	54.5
Pattern of recurrence		
Local only	9	40.9
Regional only	3	13.7
Local and regional	5	22.7
Distant with loco-regional	5	22.7
Surgery at time of recurrence		
Yes	1	4.8
No	21	95.2
Chemotherapy at time of recurrence		
Sequential	10	45.5
Concurrent	3	13.6
None	9	40.9
Re-irradiation interval (months)		
Median (range)	13 (3–1360)	-
<6 months	4	18.2
≥6 months	18	81.8
Purpose of re-irradiation		
Salvage	15	68.2
Palliation	7	31.8
Site of re-irradiation		
Primary site	10	45.5
Pelvic lymph node	7	31.8
Primary site with pelvic lymph node	5	22.7
GTV (cm^3^)		
Median (range)	99.8 (7–226.5)	-
<50 cm^3^	9	40.9
≥50 cm^3^	13	59.1
Dose of re-irradiation (EQD2, Gy)		
Median (range)	50 (26–56)	-
<50 Gy	10	45.5
≥50 Gy	12	54.5
Cumulative dose (EQD2, Gy)		
Median (range)	110 (83–148)	-
<110 Gy	11	50.0
≥110 Gy	11	50.0

GTV, gross tumor volume; EQD2, equivalent doses in 2 Gy/fraction.

**Table 3:** Disease and treatment characteristics after recurrence.

	Local Progression-Free Survival				Overall Survival			
Variable	Univariate		Multivariate		Univariate		Multivariate	
	HR (95% CI)	*p*-Value	HR (95% CI)	*p*-Value	HR (95% CI)	*p*-Value	HR (95% CI)	*p*-Value
Age (<50 vs. ≥50)	1.11 (0.38–3.25)	0.853			2.07 (0.79–5.41)	0.140		
ECOG (0/1 vs. 2)	1.49 (0.77–2.90)	0.233			2.14 (1.13–4.05)	0.019		
Histology (SCC vs. adenocarcinoma)	1.71 (0.22–13.74)	0.600			2.71 (0.34–21.72)	0.348		
Initial FIGO stage (I/II vs. III/IV)	0.94 (0.29–3.02)	0.914			0.95 (0.37–2.47)	0.918		
Progression-free interval (<10 vs. ≥10)	0.52 (0.18–0.62)	0.227			0.84 (0.34–2.07)	0.699		
SCC antigen (<1.55 vs. ≥1.55)	0.64 (0.13–3.17)	0.581			0.26 (0.06–1.10)	0.067		
CTx. at recurrence (yes or. no)	1.01 (0.57–1.79)	0.966			0.73 (0.43–1.23)	0.240		
Re-irradiation interval (<6 vs. ≥6)	0.25 (0.07–0.85)	0.026	0.11 (0.02–0.60)	0.010	0.86 (0.28–2.65)	0.795		
Purpose of RT (salvage vs. palliation)	1.04 (0.58–1.87)	0.896			1.16 (0.72–1.87)	0.536		
Presence of symptom (yes vs. no)	2.52 (0.83–7.62)	0.101			1.42 (0.56–3.64)	0.459		
GTV (<50 vs. ≥50)	5.22 (1.37–19.79)	0.015	7.31 (1.40–38.22)	0.018	8.86 (2.28–34.45)	0.002	8.71 (2.11–35.88)	0.003
Lymphatic irradiation (yes vs. no)	0.28 (0.06–1.28)	0.100			0.30 (0.08–1.04)	0.059		
Re-irradiation EQD2 (<50 vs. ≥50)	0.61 (0.21–1.76)	0.355			0.82 (0.33–2.08)	0.681		
Cumulative EQD2 (<110 vs. ≥110)	1.63 (0.54–4.91)	0.381			0.64 (0.26–1.61)	0.346		
Response (CR/PR vs. SD/PD)	0.12 (0.03–0.41)	0.001	0.28 (0.07–1.08)	0.065	0.30 (0.12–0.78)	0.014	0.35 (0.12–0.96)	0.042

ECOG, Eastern Cooperative Oncology Group; SCC, squamous cell carcinoma; FIGO, International Federation of Gynecology and Obstetrics; CTx, chemotherapy; RT, radiation therapy; GTV, gross tumor volume; EQD2, equivalent doses in 2 Gy/fraction; CR, complete response; PR, partial response; SD, stable disease; PD, progressive disease; HR, hazard ratio; CI, confidence interval.

## Data Availability

Data are available upon reasonable request.
